# 0.5 m Triboelectric Nanogenerator for Efficient Blue Energy Harvesting of All‐Sea Areas

**DOI:** 10.1002/advs.202204407

**Published:** 2022-10-17

**Authors:** Junrui Feng, Hanlin Zhou, Zhi Cao, Enyang Zhang, Shuxing Xu, Wangtao Li, Huilu Yao, Linyu Wan, Guanlin Liu

**Affiliations:** ^1^ Center on Nanoenergy Research School of Physical Science & Technology Guangxi University Nanning 530004 P. R. China; ^2^ Beijing Institute of Nanoenergy and Nanosystems Chinese Academy of Sciences Beijing 101400 P. R. China

**Keywords:** blue energy, oblate spheroid, triboelectric nanogenerators, water wave

## Abstract

Triboelectric nanogenerators (TENGs) to harvest ocean wave blue energy is flourishing, yet the research horizon has been limited to centimeter‐level TENG. Here, for the first time, a TENG shell is advanced for ocean energy harvesting to 0.5 m and an excellent frictional areal density of 1.03 cm^−1^ and economies of scale are obtained. The unique structure of the multi‐arch shape is adopted to untie the difficulty of fully getting the extensive friction layer contact. An inside steel plate is vertically placed in the center of every TENG block, which can activate the TENG to achieve complete contact even at a tilt angle of 7 degrees. The proposed half‐meter TENG (HM‐TENG) has a broad response band from 0.1 to 2 Hz, a total transferred charge quantity up to 67.2 µC, and one single TENG can deliver an open‐circuit voltage of 368 V. Coupled with the self‐stabilizing and susceptible features the ellipsoid shell brings, the HM‐TENG can readily accommodate itself to the all‐weather, all‐sea wave energy harvesting. Muchmore, the HM‐TENG is also applied to RF signal transmitters. This work takes the first step toward near‐meter‐scale enclosures and provides a new direction for large‐scale wave energy harvesting.

## Graphical abstract

In this paper, for the first time, a TENG shell is advanced for ocean energy harvesting to 0.5 m and an excellent frictional areal density of 1.03 cm^‐1^ and economies of scale are obtained. The HM‐TENG can readily accommodate itself to the all‐weather, all‐sea wave energy harvesting. Muchmore, the HM‐TENG is also applied to RF signal transmitters.

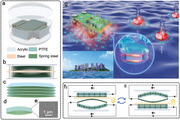

## 1 Introduction

Ocean blue energy is considered one of the most critical renewable clean energy sources,^[^ ^1^, ^2^ ^]^ and how to collect it rationally and efficiently has become the focus of researchers worldwide. It is estimated that there is more than 75 TW of energy on the surface of the oceans, and one ten‐thousandth of that would be enough to meet the needs of human life and production.^[^ ^3^, ^4^, ^5^ ^]^ If such blue energy can harvest and use effectively, it will significantly revolutionize ocean exploration, sea state monitoring, deep‐sea operation, sea‐based defense, and military fields.^[^ ^6^, ^7^, ^8^ ^]^ For the past decades, electromagnetic generator (EMG) technology has realized the collection of wave energy in the ocean. However, rare earth ore, the raw material of permanent magnets commonly used in EMG, is heavy, high cost, and easy to corrode. It may not be suitable for large‐scale collection of low frequency and random amplitude wave energy.

The emerging triboelectric nanogenerator (TENG) is an indispensable way to convert mechanical energy into electrical energy,^[^ ^9^, ^10^, ^11^, ^12^ ^]^ successfully harvests diverse kinds of mechanical energy,^[^ ^13^, ^14^, ^15^ ^]^ including wind energy,^[^ ^16^, ^17^, ^18^ ^]^ water wave energy,^[^ ^19^, ^20^, ^21^, ^22^, ^23^, ^24^ ^]^ sound energy,^[^ ^25^, ^26^ ^]^ and human movement,^[^ ^27^, ^28^, ^29^ ^]^ and has shown its value in marine‐related fields such as ship anti‐corrosion, water purification, and early warning.^[^ ^6^, ^30^, ^31^ ^]^ Over the past few years, with the hard work of more than 50 research groups worldwide,^[^ ^32^, ^33^, ^34^ ^]^ TENG has made rapid advances in output performance^[^ ^17^, ^35^, ^36^, ^37^, ^38^ ^]^ and device architecture for blue energy harvesting.^[^ ^39^, ^40^, ^41^ ^]^ Numerous structures of TENG for blue energy harvesting have been reported, including single spherical pendulum,^[^ ^42^, ^43^ ^]^ spherical folding,^[^ ^44^ ^]^ roller,^[^ ^45^ ^]^ rectangular,^[^ ^46^ ^]^ and cavity round ball.^[^ ^47^, ^48^, ^49^ ^]^ They are all uniquely constructed and improve the water wave energy collection efficiency in their way, but these devices are all ≈10 cm in size (not more than 30 cm). In contrast, the actual wave has a higher wave height (0.32–3.33 m) and longer wavelength (0.1–10 m),^[^ ^50^ ^]^ which makes it difficult for such a centimeter‐level shell of TENG to collaborate with the decimeter‐meter scale wave, leading to a low energy conversion efficiency. Research also shows a giant TENG shell can receive a higher impact force from water waves.^[^ ^51^ ^]^ However, this puzzle cannot be solved by simply scaling up conventional TENGs. Insufficient contact, low space utilization, and packaging fabrication difficulties have all become issues that limit the realization and operation of decimeter‐scale TENGs, limiting the large‐scale assembly of water‐wave energy harvesting.^[^ ^52^, ^53^ ^]^ Besides, the enormous waves caused by the changeable ocean climate put a demand on the package shape of TENG.^[^ ^43^, ^54^, ^55^, ^56^ ^]^ Generally, TENGs encapsulated in spherical, cylindrical, and cuboid shells have poor tilt resistance, which would not maintain optimal output performance or even not work if the device is capsized by huge waves, limiting energy harvesting efficiency.^[^ ^57^ ^]^ Therefore, for the large‐scale use of TENG in the ocean, we need to make targeted designs in terms of TENG size, array stacking, space utilization, and anti‐tilting.

Here, the half‐meter triboelectric nanogenerator (HM‐TENG) is first proposed for water wave energy. The short‐circuit transfer charge of HM‐TENG reaches 67.2 µC, the highest output for one water‐wave TENG. The unique structure of the multi‐arch type equipped with a vertical central steel plate is employed to boost space utilization and solve the separation problem. The large friction layers on both sides are squeezed through the movement of the steel plate to achieve complete contact and separation. The slight tilt of 7° can make the stack friction layer contact/separation from low frequency to high frequency (0.1–2 Hz) and receive good output performance. Each TENG unit has an average transfer charge of 2.4 µC (67.2 µC for 28 TENG units in HM‐TENG), a maximum open‐circuit voltage of 368 V, and a short‐circuit current of 18 µA. The flat spherical shell is designed to consist of a cylinder in the middle and two oblate spheroidal shells on the top and bottom. It is more sensitive to halcyon seas and would not lose its optimum output state even if it is overturned. Based on the ultrahigh output of the HM‐TENG, an RF signal transmitter is successfully powered, revealing its application value in the marine field. With those capacities, we expect that this work might overturn the traditional design thinking of small‐size TENGs and provide some strategies for designing and applying large‐size TENGs to realize the blue energy dream.

## 2 Result and Discussion

**Figure 1:**
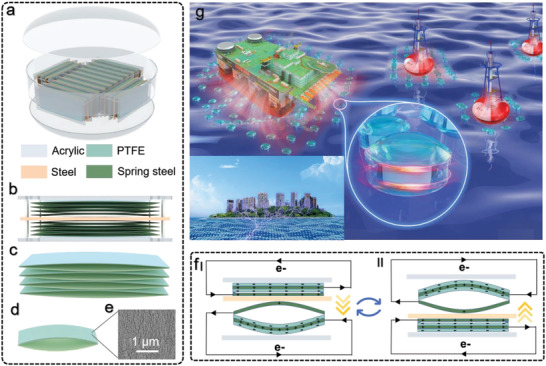
a) The exploded view of the half‐meter triboelectric nanogenerator, which is composed of 14 b) TENG blocks or 28 c) TENG units, and 112 d) TENG cells in total. e) SEM image of the PTFE surface. The scale bar is 1 µm. f) The TENG's working principle and the charge are distributed in different stages. g) Schematic illustration of the TENG array for large‐scale blue energy harvesting.

As shown in **Figure** **1**a and Figure S1 (Supporting Information), the HM‐TENG has 14 small‐size TENG blocks (Figure 1b) inside. Each block comprises two acrylic plates and a central steel plate with a TENG unit on each side. Illustrated in Figure 1c, each TENG unit is formed by 4 arched TENG cells (Figure 1d) composed of two spring steel sheets, one of which is coated with PTFE film on both sides, the other is left for exposure. The spring steel sheet has good elasticity and flatness and fatigue resistance, which makes it an ideal material for the construction of this huge TENG. The central steel plate is lifted by four slide bars penetrating four holes in the steel plates. The outer shell of HM‐TENG consists of two oblate spheroidal Hemi‐shells with a size of 50 cm and a cylindrical shell with an inner diameter of 0.5 m (Figure S2, Supporting Information). The detailed production process is explained in the experimental section. Figure 1e shows an SEM scan micrograph of the PTFE film with a scale bar of 1 µm, implying its high specific surface area and high surface charge density it might bring to the TENG device.

The HM‐TENG works in the traditional contact‐separation mode, triboelectrification and electrostatic induction are the basic principles of this kind of TENG. Specifically, PTFE films have strong electronic gain capabilities beyond metallic materials. The electrons are transferred on the surface of the PTFE when it comes into contact with the steel plate. The steel sheets and PTFE films have an equal heterogeneous charge after long‐term operation. The change in electrode distance causes the potential difference between the two electrodes, driving the flow of electrons from one electrode to the other and thus generating an external alternating current. To be more specific, the starting position of the downward movement of the steel plate is displayed in Figure 1f I. For the upper TENG cell, as the bared steel sheet leaves the PTFE film, the steel sheet coated with PTFE films would gain a more positive charge to balance the potential difference. Thus electron flows through the external circuit as the black arrow depicts. Meanwhile, the lower TENG cell would do an inverse motion, resulting in a reverse current. Followed by the steel plate moving upward as Figure 1f II presents, the bared steel sheet in the upper TENG cell contacts PTFE film has a lower electric potential which expels electrons to the steel sheet coated by PTFE film. Reverse electrons flow is received in an external circuit. The lower TENG cell also has the opposite behavior to the upper TENG cell in the above process. The up‐and‐down reciprocating motion of the central steel plate would output alternating current in an external circuit. Figure S3 (Supporting Information) shows the schematic diagram and Comsol simulation diagram of the upward and downward movement process more completely. In Figure 1g, we present a scenario for the application of HM‐TENG in the sea. HM‐TENG can provide power around large offshore base stations and detection buoys to meet the needs of long‐term work. It is even laid in the sea around the island to provide electricity for the electrical appliances on the island.

**Figure 2:**
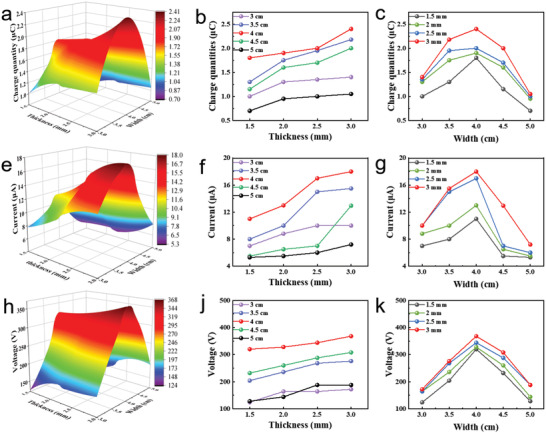
Electric characterization of the TENG on the six‐DOF motion platform. 3D surface graph of the a) transferred charge quantity per cycle, e) output short‐circuit current, and h) open‐circuit voltage versus both the steel plate thickness and device width. b,c,f,g,j,k) Corresponding 2D graphs derived from the 3D surface graph.

First, we discuss the electrical performance of the TENG block under various parameters. We placed TENG blocks with different steel plate thicknesses and device widths on a hexagonal motion platform respectively (Figure S4, Supporting Information) to measure the electrical output at a tipping angle of 15° and 0.5 Hz. This width refers to the length between the outer sides of the two acrylic sheets of the TENG block (Figure 1b). The platform can perform left‐right oscillation experiments. The electrical output performance of the TENG block is measured at a tilt angle of 15° and a frequency of 0.5 Hz. As shown in **Figure** **2**, the data is drawn into a 3D graph and smoothed with a bilinear interpolation algorithm. Two 2D plots corresponding to each 3D plot show the output performance from different views. As shown in these pictures, the transferred charge, short‐circuit current, and open‐circuit voltage increases significantly with the increase in thickness of the steel plate. Because the increase in the weight of the steel plate(Figure S5, Supporting Information)increases the mechanical energy at a certain height drop, thereby improving the electrical output performance. As the width of the device increases, the output increases first and then decreases, and the best performance all appears at 4 cm. The reason for this behavior is that when the width is ≤4 cm, the steel sheets in the TENG cell are not fully separated; when the width is greater than 4 cm, the two steel sheets are not completely in contact before entering the next cycle. In Figure 2f, a different trend from the other cases is shown due to the combined effect of the speed of the steel plate and the amount of charge under different conditions. Such changes are understandable. In Figure 2j, the variation of the potential difference is reduced due to the decrease in the separation distance of the friction layers as the thickness of the steel plate increases. In addition, the standard deviation of each value of current is between 0.4 and 0.8 µA, the standard deviation of each value of voltage is between 3–5 V, and the standard deviation of each value of the charge is between 0.05–0.1 µC. To sum up, the optimal steel plate thickness of the TENG unit is 3 mm, and the width is 4 cm, while the transferred charge per cycle, the open‐circuit voltage, and the short‐circuit current of one TENG cell are 2.4 µC, 368 V, and 18 µA, respectively. Therefore, the maximum transferred charge per cycle for HM‐TENG can reach 67.2 µC as calculated from **Figure** **4**f.

**Figure 3:**
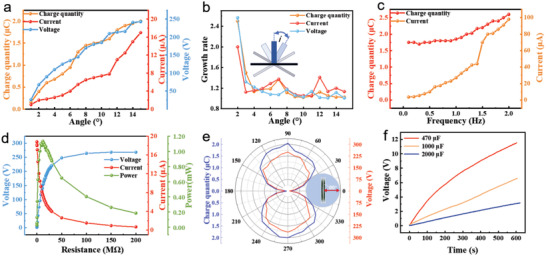
a) Electrical performance of TENG at different overturning angles. b) Electrical growth rate of TENG per degree increase in overturning angle. c) Electrical performance of TENG at different frequencies at a tipping angle of 10°. d) Output voltage and current, and output power under different external loads. e) Electrical output when TENG and motion direction is at different angles. g) Voltage curves of a different capacitor charged by the HM‐TENG under the same conditions.

Next, in **Figure** **3**, we investigate the output performance of the TENG unit under various conditions, including excitation angle, excitation frequency, matching impedance, and rotation angle. As depicted in Figure 3a, with the fixed excitation frequency of 0.5 Hz, the open‐circuit voltage, short circuit current, and transferred charge rise monotonically as the excitation angle increases. Figure 3b is a modification of Figure 3a. The vertical coordinates stand for the ratio of the output value at a current angle to that of the prior angle. In another word, Growth rate = value i° /value i°‐1°, that i° represents the current excitation angle. We propose a concept of activation angle, which is an angle when the output of the TENG is distinctly boosted compared to the prior angle in some limited scope (here is 3°–15°). The purpose of the activation angle is to evaluate the tilt angle of the TENG device when it starts to work efficiently. When the output ratio of angle i° to angle i°‐1° is the first extreme value, i° is the active angle. Although the purpose of TENG is to maximize power at the maximum tilt angle, the TENG motion in ocean waves does not always work at the maximum tilt angle. TENG works more between the activation angle and the maximum tilt angle. The smaller activation angle means that the effective tilt angle and effective working time of the TENG device are increased to harvest more energy. In future practical applications, the health of TENG devices can be judged by detecting this index. If the activation angle increases with the use time of the TENG, it should be replaced with a new TENG device in time for the work. Therefore, the concept of activation angle is very useful to evaluate TENG devices for ocean wave harvesting. For a TENG working contact‐separation mode, the smaller the input activation angle, the better energy harvesting capability the TENG has. As illustrated in Figure 3b, the times have an extreme value at 7°, which is the activation angle of HM‐TENG, implying its superior capacity in converting water wave energy at halcyon sea to electric energy. In the figure, poles appear at 7° and 12° respectively. When the inclination angle is 7°, the falling of the steel plate greatly increases the contact area of the friction layer, which leads to the appearance of the extreme value. When the angle reaches 12°, the increase in the falling speed of the steel plate leads to the appearance of the extreme value. Figure 3c shows the increased transfer charge, and short‐circuit current with increasing frequency (0.1–2 Hz, with steps of 0.1 Hz) for a TENG unit with an excitation angle of 10° (Figure S6, Supporting Information). As the frequency becomes faster, the mechanical energy of the steel plate increases, thereby improving the transfer charge and current of the TENG unit. Investigation of the output performance of HM‐TENG under different load resistances is important for practical applications. With an excitation angle of 15° and a frequency of 0.5 Hz, the output current, voltage, and power under different load resistances for HM‐TENG are shown in Figure 3d. As the external load resistance increases, the current falls, and the voltage rises while the instantaneous output power initially increases and then decreases, reaching a maximum of 1.13 mW at a load of 12 MΩ. Under the highest power load of 12 MΩ, the power density of HM‐TENG is 2.44 Wm^−3^, which is calculated by the following formula:

(1) $$P_{d}=\frac{2*P_{T}}{V_{T}}$$

*P*_T_ is the maximum power of the TENG unit, and *V*_T_ is the volume of the TENG block. A TENG block consists of two TENG units. The energy conversion efficiency is 0.83％, which is calculated by the following formula:

(2) $$\eta =\frac{W_{\mathrm{out}}}{W_{\mathrm{in}}}$$

*W*_out_ is the output energy under a 12 MΩ load, which is 149.8 µJ (Figure S7, Supporting Information) in a half cycle. This is because one steel plate drives two TENG units. The win is the change in mechanical energy of the steel plate moving 15 mm at a 15° inclination angle, and this value is 17.99 mJ. The volume energy density is 583.6 Jm^−3^, which is calculated by the following formula:

(3) $$E_{d}=\frac{W_{\mathrm{out}}*N}{V_{T}}$$

*V*_T_ is 924 cm^3^, which is the volume of the TENG block. The value of *W*_out_ is the same as above. N is 3600, which is the number of half cycles in an hour. Figure 3e shows the output of the TENG unit at different angles to the excitation direction (Figure S8, Supporting Information). The hexagonal motion platform runs with an overturning angle of 15° and 0.5 Hz. As shown in the schematic diagram, the red bidirectional arrows represent the movement direction of the platform. When the angle is 90°, the output of the TENG unit is the highest. When the angle is 30°, the TENG unit has 25% of the maximum output. The energy harvesting of the TENG unit has directional limitations. The angle between the TENG unit and the excitation direction ranges from 30°–150°. Despite the poor directional response of one TENG block, the HM‐TENG can improve the energy conversion efficiency in all directions by stacking the TENG blocks vertically. To further verify the energy harvesting capability of HM‐TENG, 470 µF, 1000 µF, and 2200 µF capacitors are charged respectively by HM‐TENG excited in a wave‐making pool (excitation frequency of 1.25 Hz), the charging voltage curves are depicted in Figure 3f. 0.47 mF capacitor can be charged to 11 V after 10 min, while the 1 mF capacitor is charged to 6 V. HM‐TENG Charges the 2.2 mF capacitor to 3 V for 600 s.

**Figure 4:**
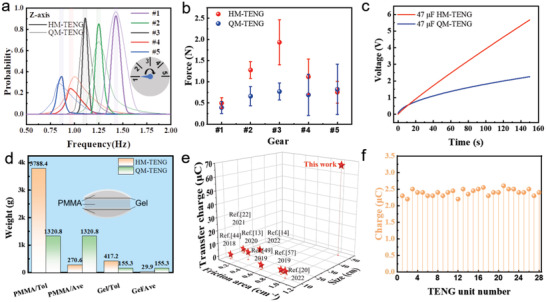
The comparison between HM‐TENG and QM‐TENG in a) the frequency distribution under different excitations, b) the force under different excitations (mean ± SD, *n* = 3, *p < 0.05, one‐way ANOVA), c) the charging performance of the same TENG block under the same excitation, and d) the packaging materials. e) Comparison of HM‐TENG with the previous water wave TENGs. f) Charge transfer amount of 28 TENG units under a 15‐degree overturning angle and 0.5 Hz frequency excitation.

**Figure 5:**
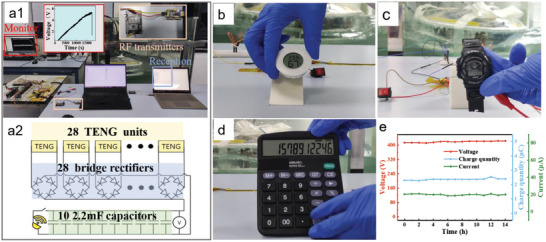
a1) Photo of a practical application of HM‐TENG to power an RF module, which is driven by the HM‐TENG through a2) the circuit diagram of the external load. Demonstrate HM‐TENG as a power source for b) a temperature and humidity sensor, c) an electronic watch, and d) a calculator. e) Durability performance of TENG.

Large‐scale TENG devices for harvesting water energy might have strong potential in the vast ocean, providing a cost‐effective route for TENG industrialization. To demonstrate this, we fabricated a quarter meter TENG (QM‐TENG) with a similar profile (Figure S9, Supporting Information), only reduced the case size to 25 cm, encapsulated one TENG block, and counterweighted it to have the same depth in water as the HM‐TENG. To find out the interaction between these two sizes of TENG and water waves, a small and lightweight inertial test unit (BEWIS SENSING, BW‐IMU500C) was placed in the center of the two TENGs to collect motion data in three‐axis acceleration (ax, ay, az). A wave pool (1.2 meters long, 1 meter wide, and 1 meter high) is applied for all the water tests throughout the whole paper. Thirty high‐power wave pumps (50 W each) are installed inside the pool (3*10 horizontal arrangement) to generate adjustable waves (Figure S10, Supporting Information). All wave pumps work at the same phase, frequency, and power through the main switch, during the experiment. The IMU can collect motion data under different switch gears (#1 – #5). The frequencies of the water waves generated in the water tank by the five gears (#1 – #5) of the water pump are 1.4 Hz, 1.2 Hz, 1.1 Hz, 0.9 Hz, and 0.8 Hz, respectively. The heights of the water waves are 4 cm, 5 cm, 6 cm, 5 cm, and 4 cm, respectively. Compared with the wave data simulated by Froude's similarity law in the TENG behavior experiment in real ocean waves (height 6 cm, frequencies 0.9, 1.1, and 1.4 Hz), the wave conditions (height 6 cm, frequency 1.1 Hz) in Figure 4c and Figure 5 conform to Froude's similarity law.^[^ ^58^ ^]^Every test for each gear last for 10 min. After data preprocessing, the average acceleration and the standard deviation of the acceleration in 10 min can be calculated by the formula:

(4) $$a_{t}=\sqrt{a_{x}^{2}+a_{y}^{2}+a_{z}^{2}}$$

(5) $$\bar{a}=\frac{\sum_{t=1}^{n}a_{t}}{n}$$

(6) $$S=\sqrt{\frac{\sum_{t=1}^{n}{\left(a_{t}-\bar{a}\right)}^{2}}{n}}$$

In the above formula, *a_t_* represents the acceleration at that moment, *a_x_*, *a_y_*, *a_z_* represent the acceleration of the three axes at this moment. $\bar{a}$ is the average acceleration over time. *S* is the standard deviation of the acceleration. On the other hand, we also calculate the frequency distribution curve from the data of *a_z_*. The calculation process includes data point sampling, distribution statistics analysis, frequency distribution curve fitting, and kernel acceleration data extraction. Then we discuss the comparison results of HM‐TENG with QM‐TENG and other TENG devices. First, data point sampling aims to search for the position of the wave crest or wave trough on an acceleration curve, and the time difference between adjacent wave crests representing different motion periods of the floating body, and the reciprocal is the frequency. By counting the frequencies within the frequency interval, we obtained the frequency distribution curve in Figure 4a, representing the motion frequency distribution of the corresponding TENG. This type of curve reflects the frequency distribution of motion within a certain period under specific conditions. Under different excitation frequencies (switch gears #1 – #5), the maximum probability response frequencies of HM‐TENG and QM‐TENG are similar. This indicates that the TENG package size does not significantly affect the response frequency to waves. Among them, the frequency distribution of 0.9 Hz corresponding to gear #4 is asymmetrical. Because under a limited distance, the energy of the water waves generated by the wave pump is not exhausted. Weak water waves are reflected from the pool wall so that the floating body receives a frequency slightly higher than the excitation frequency of the current wave pump in a short period. This phenomenon also occurs at other excitation frequencies. At 0.9 Hz, the reflected water wave has a more significant effect on the newly generated water wave. The mean value and standard deviation of the force of HM‐TENG and QM‐TENG are shown in Figure 4b. Benefiting from the larger bottom area, the average net force of the HM‐TENG is larger than that of the QM‐TENG except for the #5 gear with a lower value. This indicates that the large‐scale TENG with a larger surface area can receive more force from water waves and capture more energy.

The comparison of energy harvesting capabilities is important and necessary. The charging test is also carried out to further demonstrate the superiority of large‐scale TENG. Figure 4c compares the output of the same TENG block inside the two TENGs while they are charging a 47 µF capacitor. The TENG block is placed at the center position of the two TENGs respectively while maintaining the same draft height by adding extra counterweights. It can be seen from Figure 4c that the energy extraction efficiency of HM‐TENG is much higher than that of QM‐TENG. After 150 s, the capacitor can be charged to 5.5 V by the TENG block in HM‐TENG while 2 V for that in HM‐TENG. The energy harvesting efficiency of the TENG block is increased by 175%. Although the size of the wave pool is smaller compared to HM‐TENG and is affected by the reflection of water waves, these do not affect our experimental results and conclusions. Because it can be seen from Figure 4a that HM‐TENG and QM‐TENG are mainly excited by nascent waves; Figure 4b shows the advantage of the large size of HM‐TENG; Figure 4c shows the energy harvesting advantage brought by the large size and volume.

Besides the energy conversion efficiency, the cost is another critical issue when constructing a TENG array to harvest blue energy. We also demonstrate that large‐scale TENGs can have better economies of scale. Acrylic and silica gel are the main materials for TENG packaging. Acrylic is used as the TENG shell to make it float on the sea to harvest ocean energy. Silicone is used to seal the gap between the acrylics to prevent water from damaging the TENG. The economies of scale are evident in the packaging of large‐scale TENGs. As shown in Figure 4d, the HM‐TENG package uses 3788 g acrylic acid and 417 g silica gel. The QM‐TENG package used 1320 g acrylic and 155 g silicone. When the encapsulation material is averaged on each TENG block, HM‐TENG uses 270 g acrylic and 29 g silicone. As can be seen from Figure S11 (Supporting Information), the HM‐TENG package uses 0.099 g acrylic and 0.01 g silicone per cubic centimeter. In comparison, 40% acrylic and 60% silicone are saved. We can see in Figure 4e that the work is located at the maximum of each coordinate in the 3D plot. Especially in the coordinate dimension, its value is 5 times the minimum value. From the ordinate, the transfer charge of this work reaches 67.2 µC (Table S1, Supporting Information), which is 6 to 300 times that of other work. The increase in the number of transferred charges means that the HM‐TENG is successful, in terms of TENG size enlargement. This is the direction that must be developed for TENG applications. A larger size means more space in the package cavity. More space means more friction area can be achieved by stacking. More friction area allows more charge transfer. Compared with other reported small‐volume TENGs, we will transfer a larger amount of charge during the same motion cycle.

Although the transfer benefits from the large size of the HM‐TENG, the friction area density (1.03 cm^−1^) is also the highest in the HM‐TENG. The friction area density refers to the area of the friction layer per unit volume. Compared with the TENG device in Figure 4e, the charge density of HM‐TENG is 0.002 µC cm^−3^ in the second highest position. As can be seen in Table S2 (Supporting Information), the HM‐TENG possesses the highest charge density compared to the curved shell TENG device. Figure 4f shows the amount of transferred charge for each TENG unit moving on a hexagonal free platform with a tilt angle of 15° and a frequency of 0.5 Hz. The average value of the charge transferred by a TENG unit is 2.4 µC. In addition, Figure S12, Supporting Information shows the effect of the number of parallel TENG units on the electrical output, which is excited on a six‐axis platform with a tilt angle of 15° and a frequency of 0.5 Hz. The charge transfer amount and current increase with the increase of the number of TENG units, and the voltage remains ≈370 V. Furthermore, we conduct experiments on the electrical output of the TENG unit under everyday conditions. In Figure S13 (Supporting Information), the charge and voltage decrease slightly with increasing relative humidity (20–90%) at 24 °C.^[^ ^59^ ^]^ The voltage and charge remain stable with temperature (20–40 °C) at 20% RH.^[^ ^60^ ^]^

Finally, **Figure** **5** shows the practical application of HN‐TENG in a pool. With the above properties, HM‐TENG can be used as a blue energy harvesting device in principle. To test the feasibility of this application, the HM‐TENG is put into a large wave pool (1.2 meters long, 1 meter wide, and 1 meter high), (Figure 5a1 ;Figure S10, Supporting Information). 28 TENG units are connected in parallel after passing through the rectifier bridge respectively to charge the 22 mF capacitor bank (10 2.2 mF capacitors are connected in parallel). When the voltage reaches 5 V (Figure S14, Supporting Information), the switch turns on, and the RF module starts to work and sends information successfully (Video S1, Supporting Information). Figure 5a2 is the circuit diagram of the external load. To test the possibility of HM‐TENG powering island residents. Figure 5b–d shows that the HM‐TENG powers commercial electronic products such as Thermo hygrometers, electronic meters, and calculators, respectively (Video S2, Supporting Information). The excellent robustness of HM‐TENG is demonstrated in Figure 5e. Under continuous 14 h of operation (Figure S15, Supporting Information), the electrical performance did not degrade. In addition, we conducted durability experiments for 3 days, every day from 8 am to 10 pm. As can be seen from Figure S16 (Supporting Information0, there is no degradation in electrical performance after 42 h. With the development of science and technology, we envision that shortly, TENG‐based blue energy harvesting technology will have many practical applications, such as offshore marine power stations, smart fishing farms, and even aircraft carrier generators.

## 3 Conclusions

We design and manufacture HM‐TENG for the first time. It has a higher shell size, friction layer density, and transferred charge than the reported work. In addition, it shows its greater potential for water energy harvesting, stability, and economic benefits. On the one hand, the relationship between different structural parameters (plate thickness, spacing) and the output of the TENG element is discussed. When the plate thickness was 3 mm and the spacing was 4 cm, the single unit of TENG has a maximum open‐circuit voltage of 368 V, a max short‐circuits cut current of 18 µA, and the transfer charge of 2.4 µC. On the other hand, the output performance of TENG under different input conditions was studied. When the swing angle is fixed at 10°, the output of the TENG gradually increases with the increase of frequency. Under the motion frequency of 0.5 Hz, the output of TENG increases continuously with the continuous increase of the swing angle (from 1° to 15°, step size 1°). In particular, the activation angle of the TENG unit is as small as 7° (the output charge also reaches 1.6  µC), which demonstrates its high sensitivity and the feasibility of harvesting all‐weather wave energy in the whole sea area. Based on these excellent properties, HM‐TENG can drive various electronic devices, such as radio frequency modules. This study proposes a new structural design for TENGs produced in offshore power plants. This may inspire new research on large TENGs.

## 4 Experimental Section

### Production of TENG unit

A100 mm × 200 mm spring steel sheet (Kobetool, Germany, 100 mm wide and 0.08 mm thick) with a thickness of 80 µm was cut, cleaned with ethanol, and dried with an air gun. A PTFE membrane with a thickness of 80 µm was attached to both sides of the spring steel sheet. The enameled wire with a diameter of 0.2 mm is drawn out to output electric energy. The spring steel sheet with PTFE and the long side of the exposed spring steel sheet as opposed to forming a TENG, and the exposed spring steel sheet was used as the other electrode. The spring steel sheet with attached PTFE membrane (80 µm thick) and the exposed spring steel sheet form a substantially arched unit (Figure 1d). Four basic arched units were stacked together with double‐sided tape to form a TENG unit (Figure 1c).

### Shell Production

An ellipsoid with a thickness of 5 mm, a diameter of 50 cm, and a height of 7 cm, and an acrylic cylinder with a thickness of 5 mm, a diameter of 50 cm, and a height of 10 cm were customized, and a flange of 15 mm and 12 holes of 3 mm were designed. First, tighten with 20 mm long M3 screws, then use black silk waterproof tape for the first layer of sealing, and finally use Kafuter K‐704 silicone sealant (dry in 24 h). A hole is made in the upper ellipsoid shell to lead the line out.

### Test Method

For the electrical measurement of TENG, the Keithley 6514 system electrometer was used to measure the current and charge, and the MDO3012 oscilloscope was used to measure the open‐circuit voltage. The Hexagonal free platform provides uniform and variable overturning motion. The 30 wave pumps in the wave pool simulate sea wave conditions.

### Statistical Analysis

The data preprocessing included debiasing and filtering. Each experiment was repeated three times. The magnitude of the force was expressed as the mean ± standard deviation (SD). Statistical analysis was performed by using SPSS software. All results were analyzed by one‐way ANOVA, where *p* < 0.05 was considered statistically significant.

## Conflict of Interest

The authors declare no conflict of interest.

## Supporting information

Supporting Information

Supplemental Video 1

Supplemental Video 2

## Data Availability

The data that support the findings of this study are available from the corresponding author upon reasonable request.
