# Supplementary material for: 0.5 m Triboelectric Nanogenerator for Efficient Blue Energy Harvesting of All‐Sea Areas
Source: Adv Sci (Weinh). 2022 Oct 17;9(35):2204407. doi: 10.1002/advs.202204407 (PMC9762320; doi:10.1002/advs.202204407)
Supplement: Supplementary file 1 — Supporting Information [file ADVS-9-2204407-s002.pdf]

ADVANCED SCIENCE
Open Access

Supporting Information

for *Adv. Sci.*, DOI 10.1002/advs.202204407

0.5 m Triboelectric Nanogenerator for Efficient Blue Energy Harvesting of All-Sea Areas

*Junrui Feng, Hanlin Zhou, Zhi Cao, Enyang Zhang, Shuxing Xu, Wangtao Li, Huilu Yao, Linyu Wan and Guanlin Liu**

Supporting Information

0.5 m Triboelectric Nanogenerator for Efficient Blue Energy Harvesting of All-Sea Areas

*Junrui Feng, Hanlin Zhou, Zhi Cao, Enyang Zhang, Shuxing Xu, Wangtao Li, Huilu Yao, Linyu Wan, Guanlin Liu**

J. Feng, H. Zhou, Z. Cao, E. Zhang, S. Xu, W. Li, H. Yao, Prof. L. Wan, Prof. G. Liu

Center on Nanoenergy Research, School of Physical Science & Technology, Guangxi University, Nanning, 530004 P. R. China.

J. Feng, H. Zhou, Z. Cao, E. Zhang, S. Xu

Beijing Institute of Nanoenergy and Nanosystems, Chinese Academy of Sciences, Beijing 101400, P. R. China

E-mail: guanlinliu@gxu.edu.cn

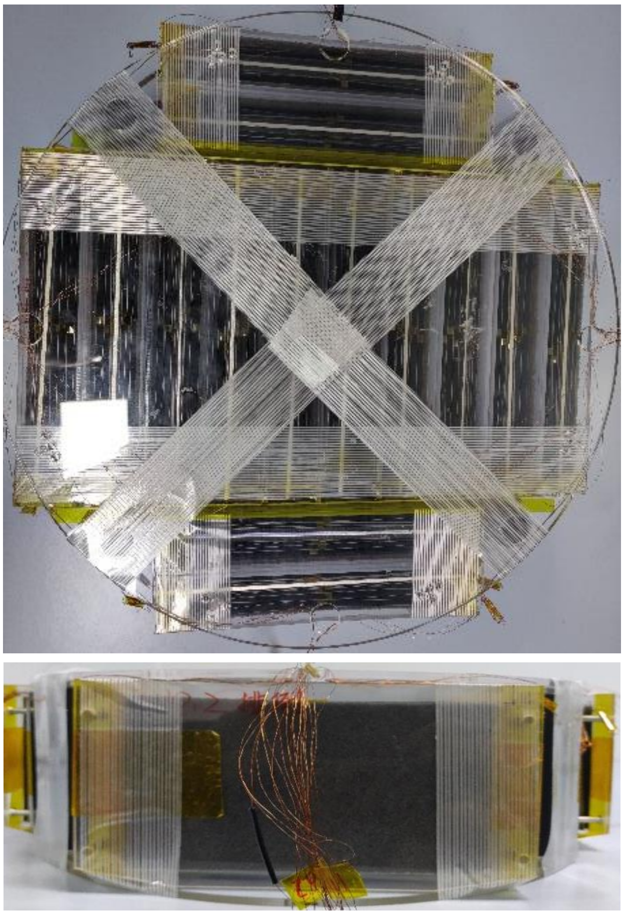

Figure S1. Actual photos of HM-TENG (top view, front view).

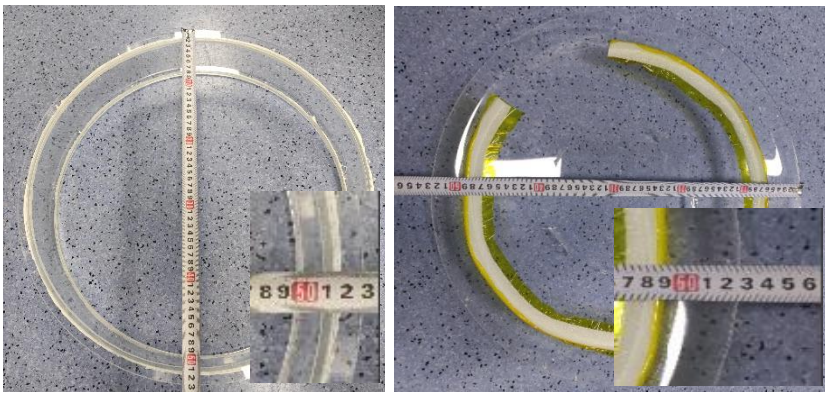

Figure S2. Case size photo of HM-TENG.

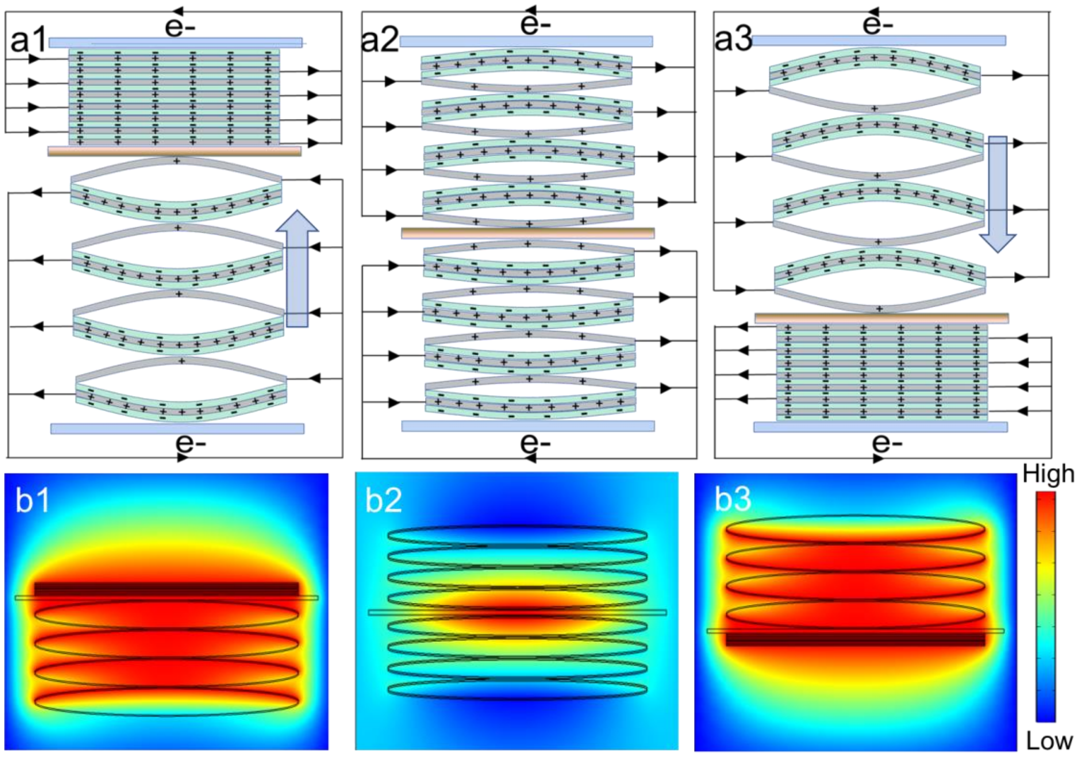

Figure S3. a) Schematic diagram of the principle of TENG, b) Simulation of the change of potential difference during the movement of TENG using COMSOL software.

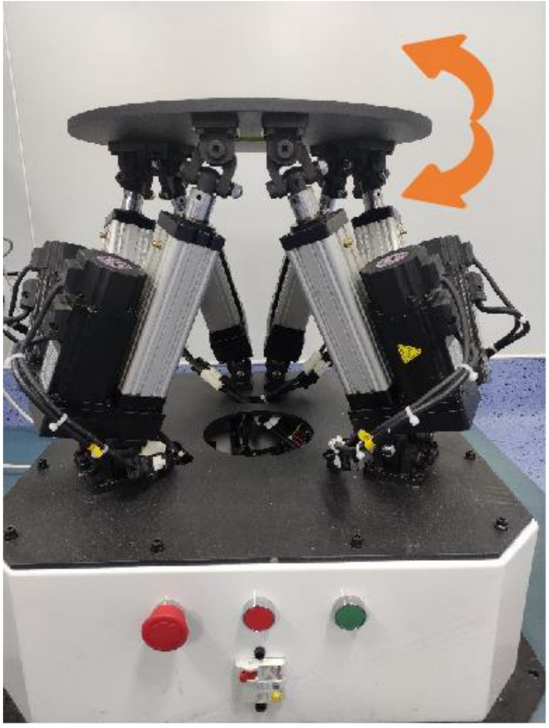

Figure S4. A hexagonal freeform platform that simulates the motion of ocean waves.

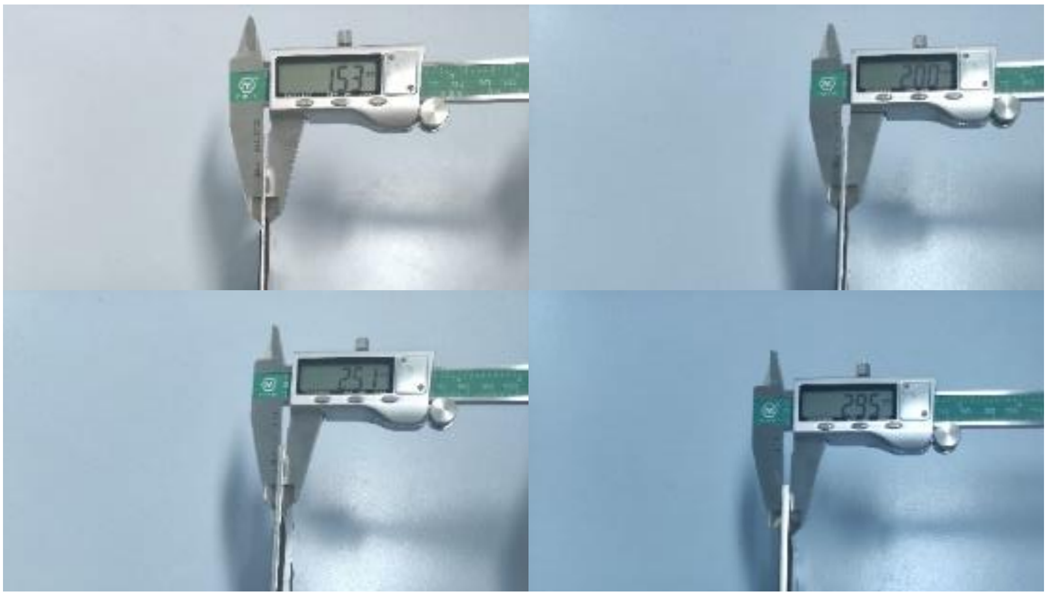

Figure S5. Steel plate (1.5mm-3mm) in the middle of the TENG block.

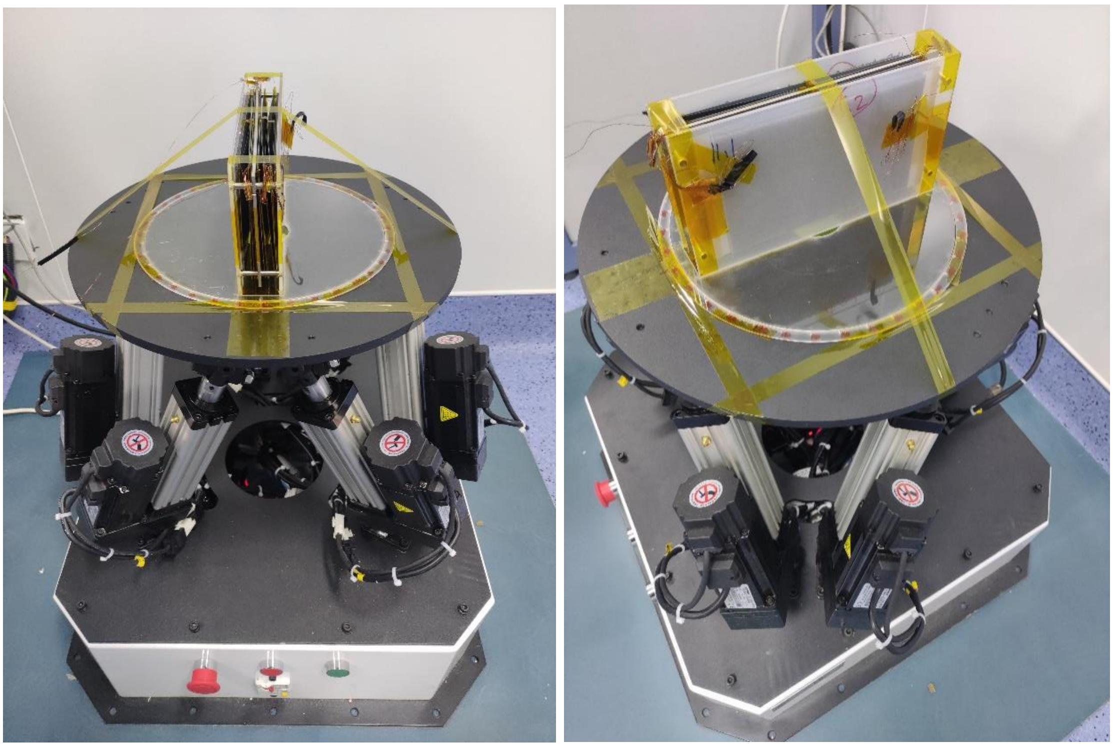

Figure S6. Picture of TENG block doing 360° rotation on a hexagonal free platform.

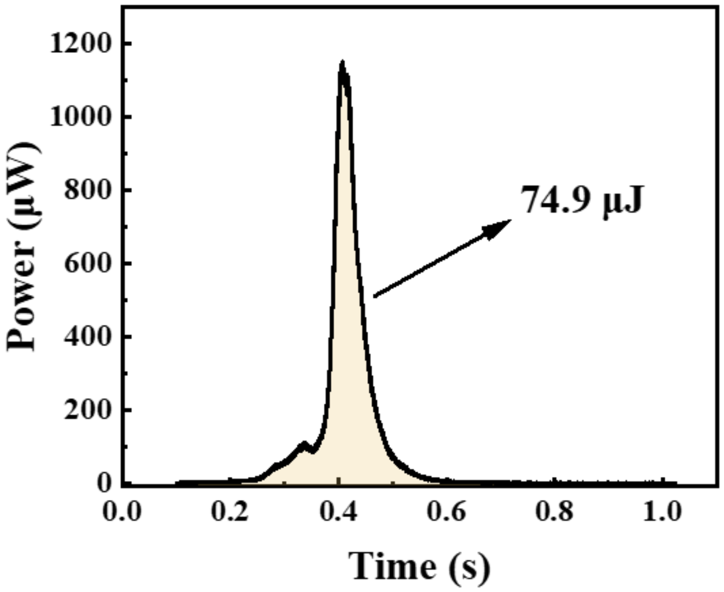

Figure S7. The energy is harvested by the TENG unit in a half cycle under a maximum power load of 12 MΩ.

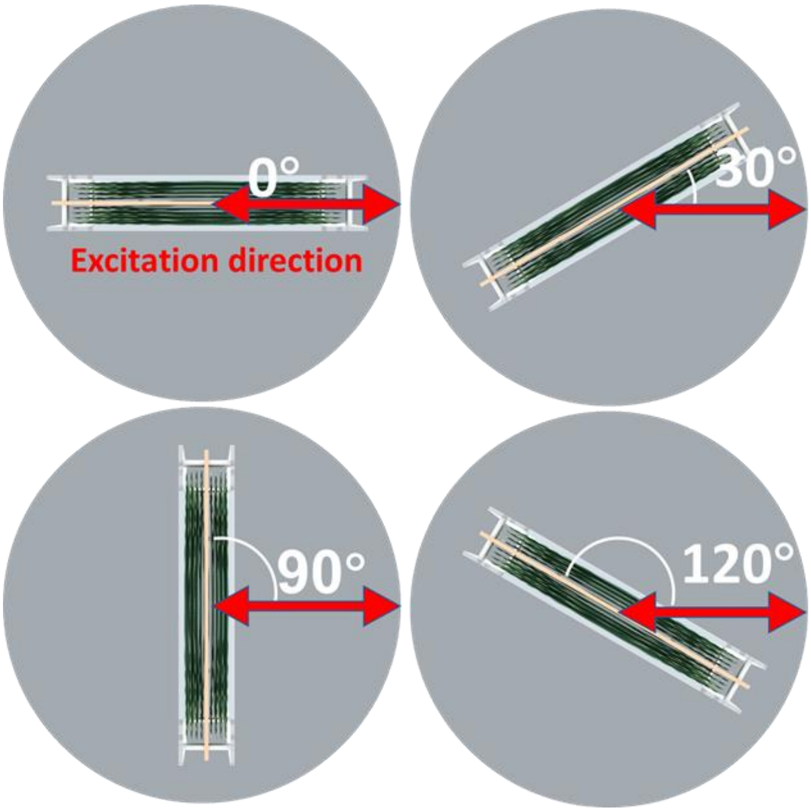

Figure S8. Schematic diagram of the angle between the TENG block and the excitation direction.

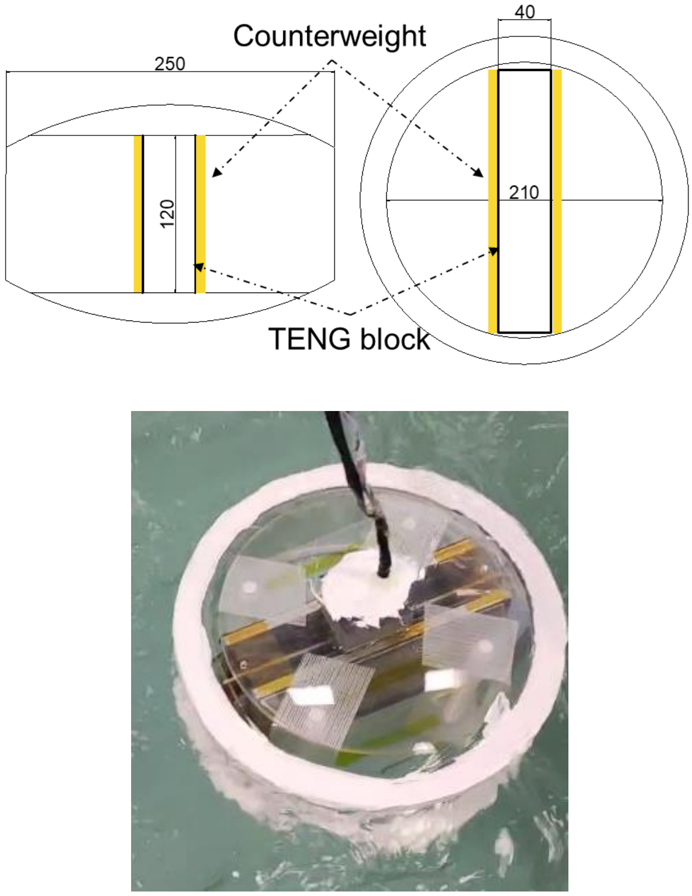

Figure S9. Physical and structural diagrams of QM-TENG.

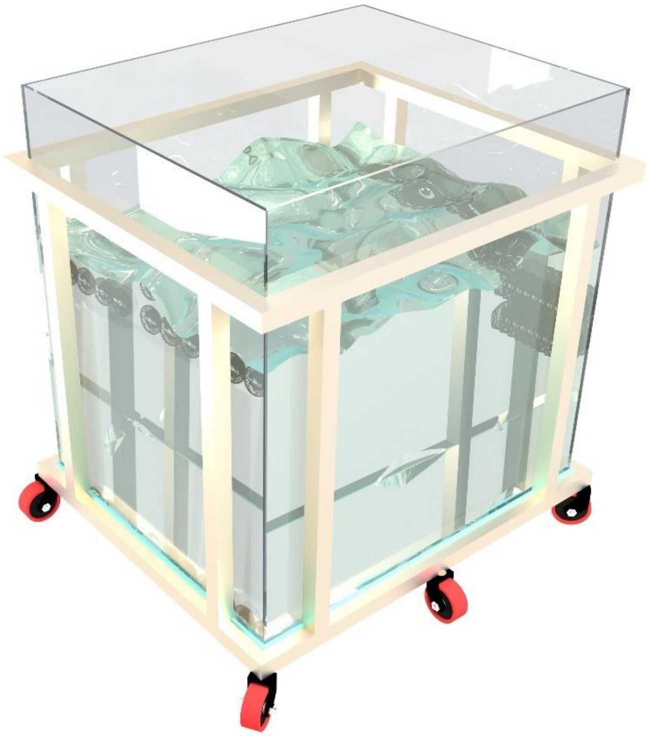

Figure S10. 3D simulation of the wave pool used in the experiment.

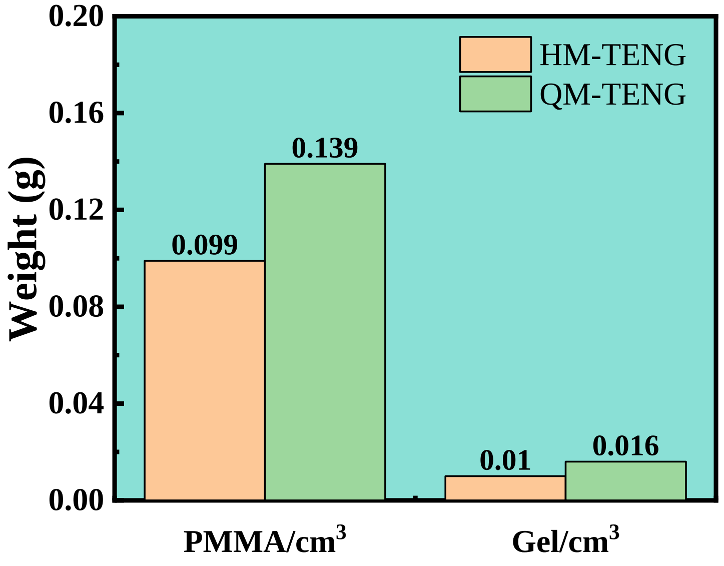

Figure S11. Comparison of HM-TENG and QM-TENG packaging materials per unit volume.

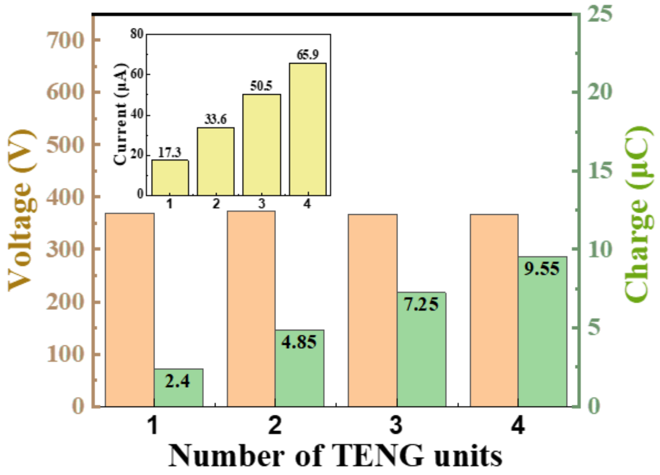

Figure S12. Influence of the number of parallel TENG units on electrical output.

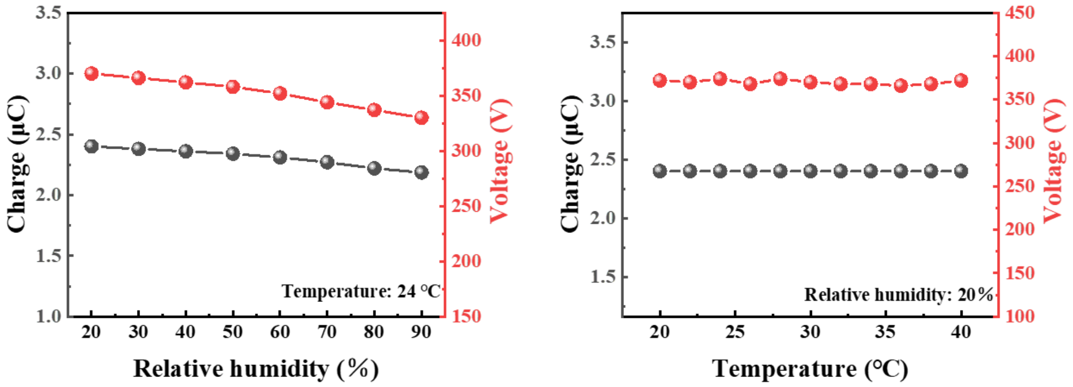

Figure S13. The electrical output performance of the TENG unit at different relative humidity and temperature.

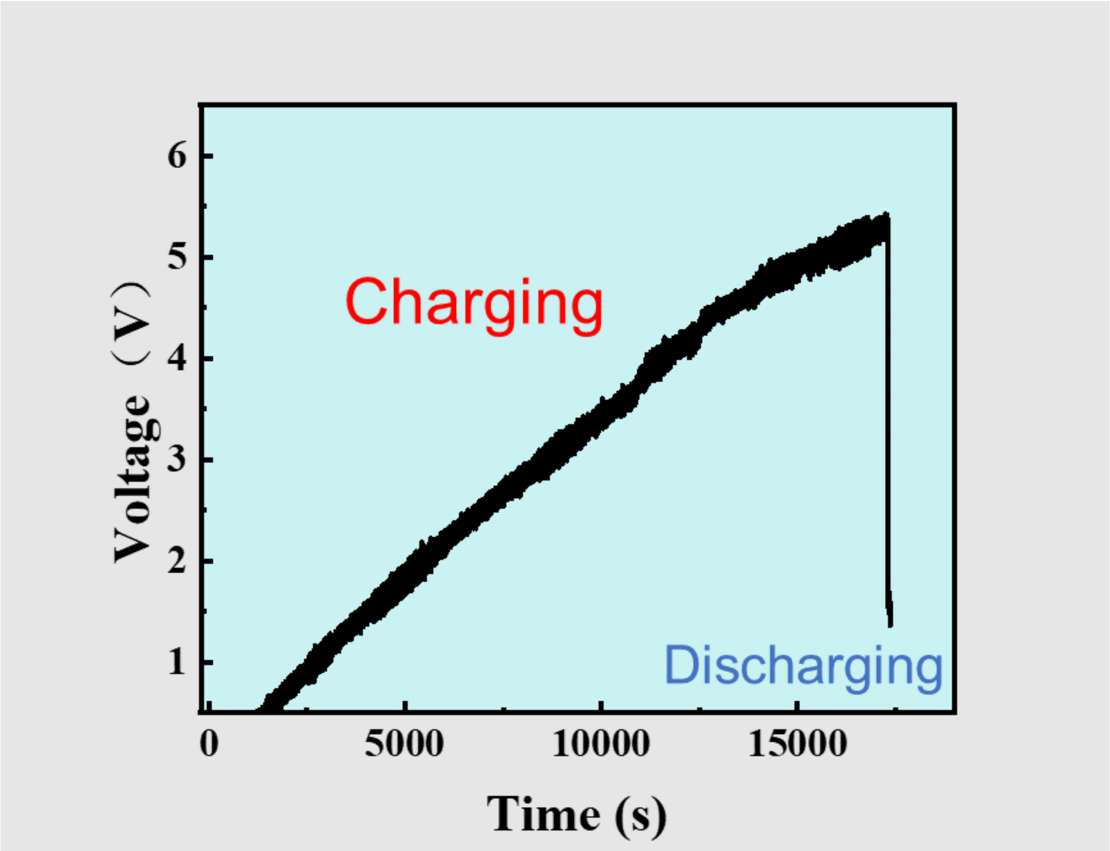

Figure S14. Capacitor voltage variation graph in RF applications monitored by electrometer 6514.

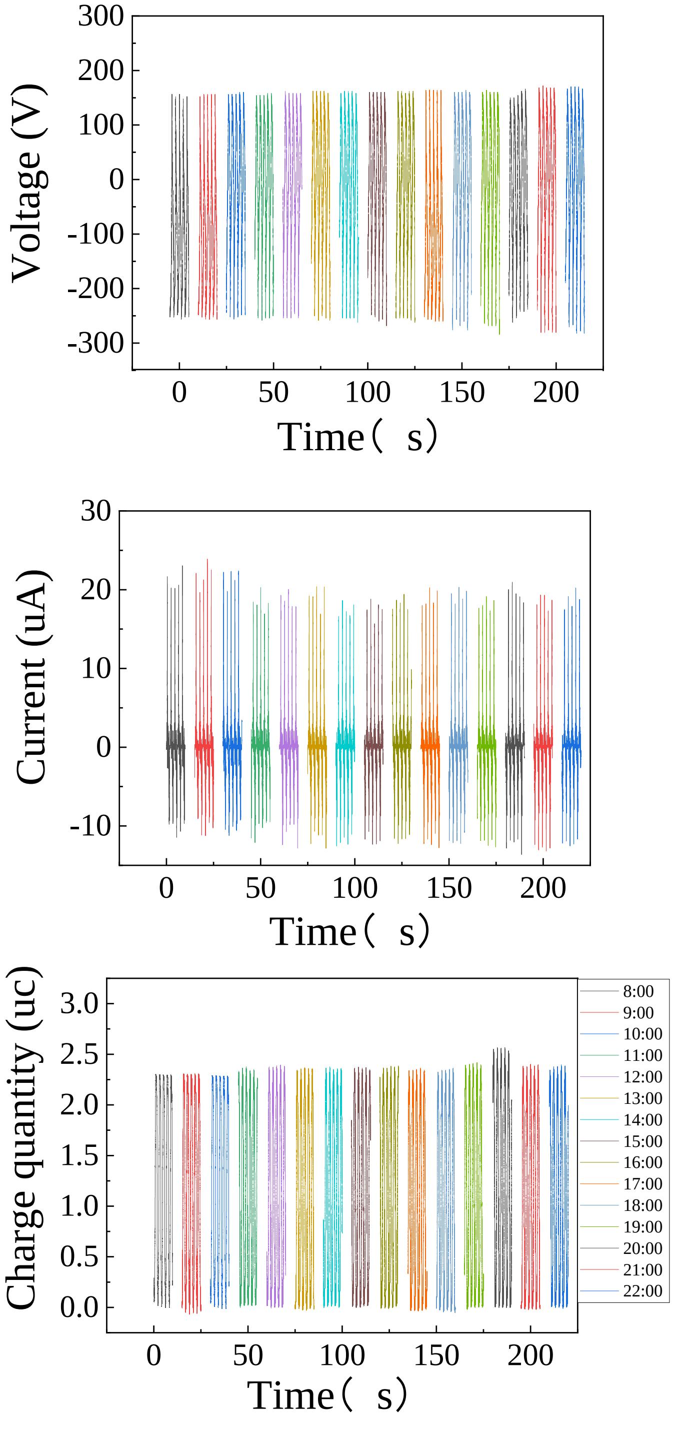

Figure S15. The voltage, current, and transferred charge of the 14-hour durability test.

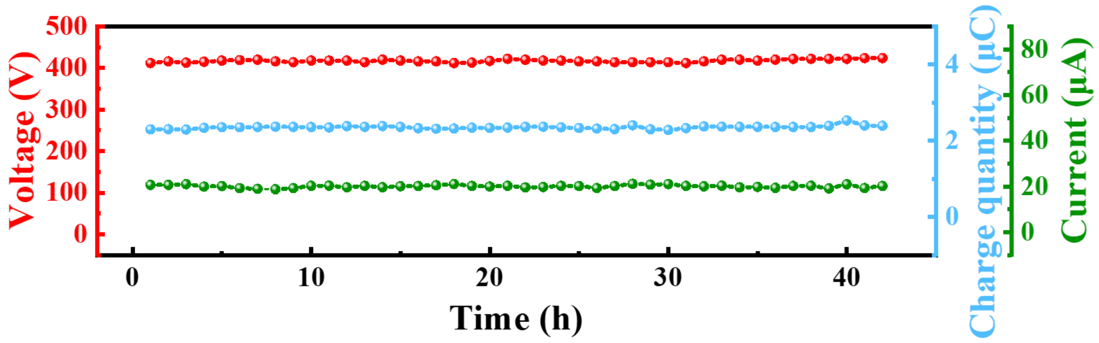

Figure S16. The voltage, current, and transfer charge of the 42-hour durability test.

Number	Charge(μC)	Number	Charge(μC)	Number	Charge(μC)	Number	Charge(μC)
1	2.3	8	2.4	15	2.45	22	2.5
2	2.2	9	2.3	16	2.5	23	2.5
3	2.5	10	2.4	17	2.55	24	2.4
4	2.4	11	2.45	18	2.3	25	2.4
5	2.4	12	2.2	19	2.4	26	2.5
6	2.3	13	2.5	20	2.4	27	2.3
7	2.3	14	2.35	21	2.6	28	2.4
Total:67.2 μC							

Table S1. Table of charge transfer amount of 28 TENG units.

Paper	Ref.[22] 2021	Ref.[57] 2019	Ref.[44] 2018.7	Ref.[14] 2022	Ref.[49] 2019	Ref.[13] 2020	Ref.[20] 2022	This work
Housing shape	cylinder	flat ball	ball	ball	ball	half cylinder	cuboid	flat ball
Charge density (μC/cm^{-3})	8.49E-05	0.00078	0.00099	0.00100	0.00114	0.00169	0.00523	0.00200

Table S2: Comparison table of charge density between HM-TENG and related TENG devices.
